# Supplementary material for: Paramecium bursaria as a Potential Tool for Evaluation of Microplastics Toxicity
Source: Biology (Basel). 2022 Dec 19;11(12):1852. doi: 10.3390/biology11121852 (PMC9775370; doi:10.3390/biology11121852)
Supplement: Supplementary file 1 [file biology-11-01852-s001.zip › biology-2054204-supplementary.pdf]

Supplementary Materials:

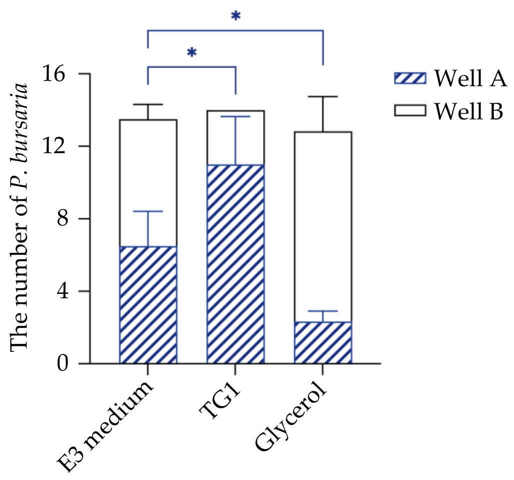

Figure S1 Analysis of prey pursuit and avoidance behaviors using customized two-well testing apparatus. To validate this testing apparatus detailed in Figure 3D, TG1 (food source of *P. bursaria* and served as attractants), 2.5% glycerol (toxic to *P. bursaria* and served as repellents) or E3 medium alone (i.e., control group) was added to well A and untreated *P. bursaria* were placed in canal C, respectively, and then dividers were carefully removed to allow free movement of *P. bursaria*. After 10 min, the number of *P. bursaria* in well A and B was counted. The data are represented as the mean ± SD of three independent experiments and asterisks above the line denote significantly different (*P <0.05) between the mean (i.e., mean number of cells in well A) identified by the line.
